# Supplementary material for: Delta Variant of SARS-CoV-2 Replacement in Brazil: A National Epidemiologic Surveillance Program
Source: Viruses. 2022 Apr 20;14(5):847. doi: 10.3390/v14050847 (PMC9143796; doi:10.3390/v14050847)
Supplement: Supplementary file 1 [file viruses-14-00847-s001.zip › Table_S4.pdf]

Table S4: Lineage effect on Ct value at the national level and for each capital using a linear regression model (β). Three distinct viral targets (N, ORFab and S) and an internal process control (MS2) were analyzed. Negative β values indicate a decrease in the Ct score in the period dominated by the VOC Delta.

Capital	Number of Samples	N gene			ORF1ab gene			S gene			MS2 control		
		β	95% CI	p-value	β	95% CI	p-value	β	95% CI	p-value	β	95% CI	p-value
Belém	2749	-1.341	±1.569	<0.1	-1.443	±1.612	<0.1	-1.487	±1.585	<0.1	-0.094	±0.799	0.817
Belo Horizonte	32179	-0.813	±0.208	*<0.001*	-0.811	±0.209	*<0.001*	-1.148	±0.207	*<0.001*	-0.290	±0.110	*<0.001*
Boa Vista	227	-	-	-	-	-	-	-	-	-	-	-	-
Brasília	2337	-1.205	±0.496	*<0.001*	-1.414	±0.500	*<0.001*	-1.583	±0.494	*<0.001*	-0.592	±0.277	*<0.001*
Campo Grande	1744	-	-	-	-	-	-	-	-	-	-	-	-
Fortaleza	91	-0.099	±6.027	0.975	0.242	±6.141	0.939	1.137	±6.203	0.72	1.117	±3.236	0.5
Goiânia	2630	-0.583	±1.059	0.281	-0.611	±1.074	0.265	-0.907	±1.068	<0.1	0.113	±0.113	0.682
Macapá	435	-	-	-	-	-	-	-	-	-	-	-	-
Manaus	327	-	-	-	-	-	-	-	-	-	-	-	-
Palmas	971	-0.040	±0.040	0.948	-0.480	±1.199	0.433	-0.619	±1.189	0.308	-0.047	±0.733	0.9
Porto Alegre	3502	-0.408	±0.535	0.134	-0.504	±0.544	<0.1	-0.793	±0.537	*<0.05*	-0.542	±0.286	*<0.001*
Porto Velho	768	-1.376	±2.359	0.254	-1.533	±2.378	0.207	-1.466	±2.421	0.236	-0.029	±1.210	0.963
Rio de Janeiro	8090	-1.502	±0.336	*<0.001*	-1.708	±0.339	*<0.001*	-1.925	±0.334	*<0.001*	-0.422	±0.178	*<0.001*
Salvador	646	-	-	-	-	-	-	-	-	-	-	-	-
São Paulo	5119	-0.591	±0.706	0.1	-0.723	±0.709	*<0.05*	-0.951	±0.707	*<0.01*	-0.471	±0.349	*<0.01*
**Brazil**	**61815**	**-0.853**	**±0.147**	***<0.001***	**-0.964**	**±0.148**	***<0.001***	**-1.258**	**±0.147**	***<0.001***	**-0.387**	**±0.077**	***<0.001***

'-' There was no lineage transition in the evaluated period; Statistically significant values are in italics ($\alpha = 0.05$);
