# Supplementary figures and images for: Delta Variant of SARS-CoV-2 Replacement in Brazil: A National Epidemiologic Surveillance Program
Source: Viruses. 2022 Apr 20;14(5):847. doi: 10.3390/v14050847 (PMC9143796; doi:10.3390/v14050847)

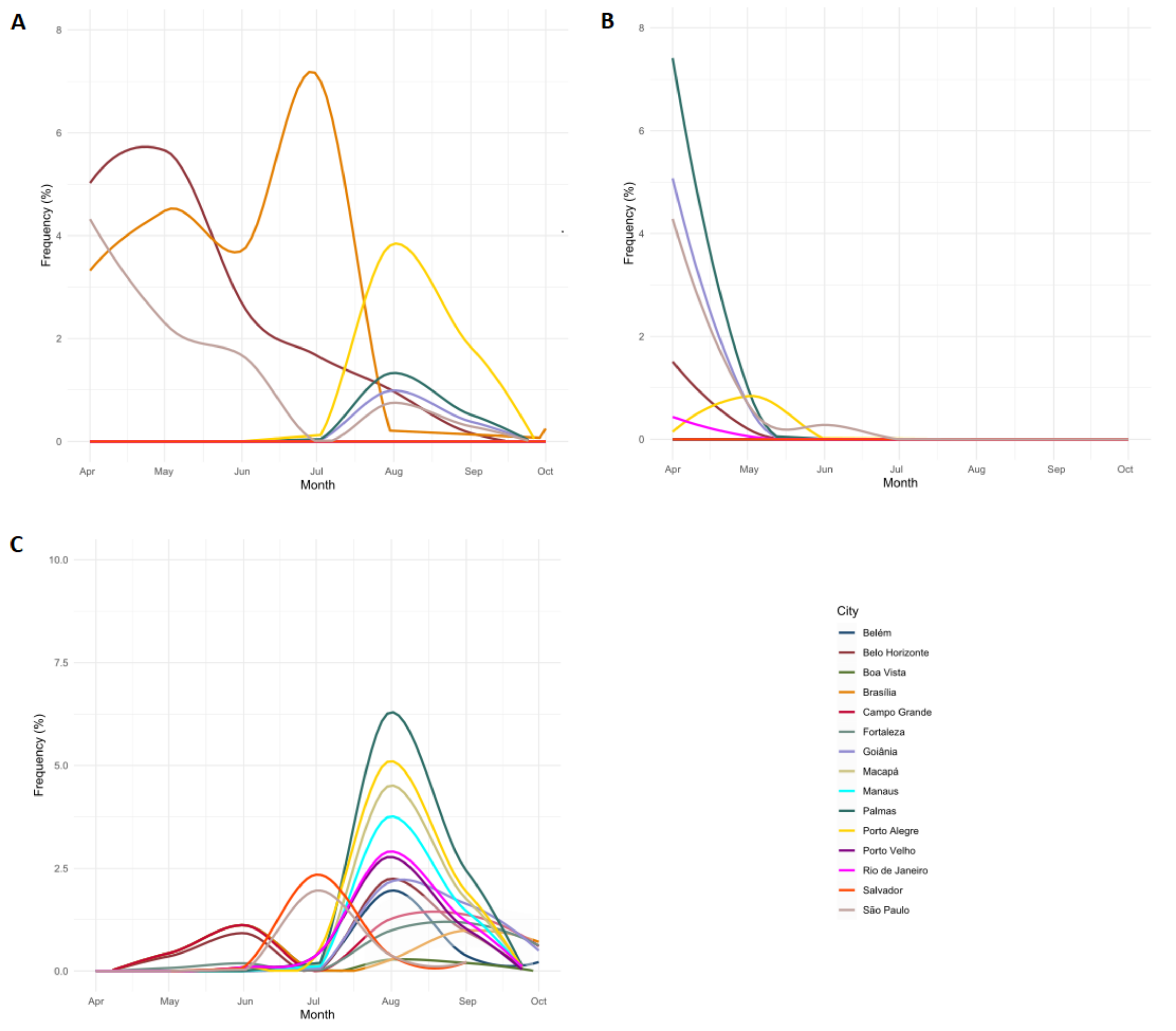

Supplement: Supplementary file 1 [file viruses-14-00847-s001.zip › Figure_S1.tif]
