# Supplementary material for: Delta Variant of SARS-CoV-2 Replacement in Brazil: A National Epidemiologic Surveillance Program
Source: Viruses. 2022 Apr 20;14(5):847. doi: 10.3390/v14050847 (PMC9143796; doi:10.3390/v14050847)
Supplement: Supplementary file 1 [file viruses-14-00847-s001.zip › File_S1.zip!/File_S1/Figure_1A/Dataset.docx]

Number of samples and representative percentage by each capital

| Region | Capital | Number of samples (n) |
| --- | --- | --- |
| North | Belém | 297 |
|  | Boa Vista | 50 |
|  | Macapá | 65 |
|  | Manaus | 60 |
|  | Palmas | 288 |
|  | Porto Velho | 155 |
| Northeast | Fortaleza | 401 |
|  | Salvador | 70 |
| Midwest | Brasília | 725 |
|  | Campo Grande | 198 |
|  | Goiânia | 471 |
| Southeast | Belo Horizonte | 1,820 |
|  | Rio de Janeiro | 1,097 |
|  | São Paulo | 1,093 |
| South | Porto Alegre | 759 |
| **Total** | | **7,549** |
